# Editorial: Reviews in emotional and behavioral dyscontrol in neurological disorders

**DOI:** 10.3389/fnbeh.2024.1447287

**Published:** 2024-07-22

**Authors:** Tao Tan, Sara Palumbo

**Affiliations:** ^1^Oujiang Laboratory (Zhejiang Lab for Regenerative Medicine, Vision and Brain Health), Institute of Aging, Wenzhou Medical University, Wenzhou, Zhejiang, China; ^2^Department of Clinical and Experimental Medicine, University of Pisa, Pisa, Italy

**Keywords:** neurological disorders, psychiatric disorders, multiple sclerosis, Tourette syndrome, bipolar spectrum disorder, Parkinson's disease

Neurological and psychiatric disorders often present complex interrelations and multifaceted challenges in their diagnosis and treatment. Recent research sheds light on innovative strategies and emerging trends that could significantly impact the management of conditions such as Parkinson's Disease (PD), Tourette Syndrome (TS), Bipolar Spectrum Disorders (BSD), and Multiple Sclerosis (MS). This editorial synthesizes the findings from four key studies, highlighting the potential for integrative approaches, collaborative efforts, and personalized care in addressing these disorders.

The paper by Zhang et al. reviewed current evidence on the effectiveness of mind-body exercises in mitigating cognitive decline in PD patients. Indeed, PD, characterized by the degeneration of brain neurons, leads to motor symptoms like bradykinesia and impaired balance, as well as non-motor symptoms such as cognitive impairment. Traditional treatment often involves a combination of medications and mental training. However, the long-term use of psychiatric drugs carries the risk of severe side effects, including brain death and movement disorders. Recent research has focused on mind-body exercises as promising alternatives, such as Tai Chi, Qigong, yoga, and dance.

Mind-body exercises integrate concentration, breath control, and physical activity, promoting mental and brain health. These practices have shown potential in enhancing cognitive performance in older adults, both with and without cognitive impairments. Although the research is promising, it also highlighted the need for further studies to address existing limitations and optimize these exercises as therapeutic strategies.

The article by Yang et al. exploited bibliometrics and visual analysis of research performed on TS children from 2011 to 2021.

TS in children remains an active research area, yet collaboration among researchers and institutions appears insufficient. About 1,200 publications were first chosen in the Web of Science Core Collection (WoSCC) database and then analyzed by the CiteSpace software. Hotspots and trends of TS were critically reviewed. The analysis revealed that the United States and University College London were the most prolific contributors. However, there was limited cooperation between institutions, countries, and authors. Key research hotspots included epidemiology, comorbidities, deep brain stimulation, behavioral therapy, and basal ganglia.

This paper also emphasized the necessity for enhanced international cooperation to advance the understanding and treatment of TS. Future research should focus on psychiatric comorbidities, effective intervention measures, and early warning systems for risk factors. Strengthening collaboration could lead to more comprehensive insights and improved care strategies for children with TS.

The review by Digiovanni et al. aimed to elucidate the clinical and biological intersections between BSD and neurological disorders by exploring evidence of shared clinical and biological characteristics.

Psychiatric symptoms often precede or complicate neurological disorders, with significant overlaps observed between BSD and neurological conditions like PD, MS, autoimmune encephalitis, epilepsy, and frontotemporal dementia. Despite these overlaps, the underlying pathophysiological mechanisms remain largely unclear. The review highlighted evidence of converging factors such as ion channel dysfunction, neuroinflammation, and impaired neurotransmission. The importance of recognizing alterations that may trigger BSD symptoms before the onset of neurological diseases has been highlighted by the authors. Understanding these intersections can guide future diagnostic and therapeutic research, potentially leading to earlier and more effective interventions.

The opinion article by Palumbo and Palumbo discussed recent evidence and perspectives on the research linking social cognition deficits with MS. The research underscored the limited knowledge about social cognition deficits in MS, such as emotion recognition and theory of mind, negatively affecting the clinical management of these aspects.

Emotional dyscontrol, such as emotional blunting, uncontrollable crying and/or laughing, and episodes of irritability, sadness, and tearfulness without apparent reasons, has been shown to significantly affect the overall quality of life of many patients with MS. However, emotional dyscontrol in these patients is frequently underdiagnosed and underreported, partly due to the stigma surrounding the condition and because it has been viewed as a psychological reaction to the disease. Moreover, the lack of standardized diagnostic criteria and consensus on classification has been highlighted as an additional factor that complicates the identification of emotional dyscontrol.

This article advocated increased awareness and routine assessment of emotional dyscontrol in clinical settings to improve diagnosis and treatment. Personalized assessments and standardized evaluation tasks were recommended to better understand these deficits. Addressing social cognitive impairments could enhance the social and psychological quality of life for individuals with MS.

## Conclusions

These papers collectively underscore the need for innovative, integrative approaches to manage neurological and psychiatric disorders. Mind-body exercises offer a promising non-pharmacological treatment for cognitive impairment in PD, while enhanced collaboration and focus on comorbidities could advance TS research. Understanding the intersections between BSD and neurological disorders may lead to earlier and more effective interventions. Finally, recognizing and addressing emotional dyscontrol in MS through personalized assessments could significantly improve patient outcomes.

As research continues to evolve, fostering collaboration across disciplines and integrating personalized care strategies will be crucial in improving the quality of life for individuals affected by these complex disorders.

## Author contributions

TT: Writing – review & editing. SP: Writing – review & editing, Writing – original draft, Conceptualization.

